# The effects of qigong intervention based on the Internet on quality of life and physical fitness in Chinese postoperative breast cancer patients: a protocol of randomized controlled trial

**DOI:** 10.1186/s13063-023-07187-2

**Published:** 2023-03-13

**Authors:** Chengxiang Li, Xiaosheng Dong, Lina Yu, Kai Yuan, Xiangren Yi, Yuanlong Shen, Hu Niu

**Affiliations:** 1 College of Physical Education, Shandong Normal University, Jinan, 250014 China; 2 College of Physical Education, Shandong University, Jinan, 250011 China; 3 The First Affiliated Hospital of Shandong First Medical University, Jinan, 250011 China; 4 Department of Breast and Thyroid Surgery, Jinan Central Hospital Affiliated to Shandong First Medical University, Jinan, China

## Abstract

**Background:**

The purpose of this study is to verify the improvement of remote qigong intervention on the quality of life and physical fitness of breast cancer patients after surgery by means of a randomized controlled trial and to compare it with the conventional exercise combination of aerobic exercise and resistance training.

**Methods/design:**

The research approach applied in this study is a randomized controlled trial. After completing the baseline questionnaire and physical fitness test, participants were randomly assigned to either the qigong group or the combined exercise rehabilitation group. Patients in the qigong group performed Qigong-Baduanjin twice a week for 30 min each time under remote guidance and practiced Baduanjin by themselves at other times. Patients in the combined exercise rehabilitation group were given resistance training twice a week for 30 min under remote guidance, and walking the rest of the time. At the end of the 12-week intervention, outcomes testing and data collection were carried out. The primary outcomes are quality of life, measured using the Medical Outcomes Study 36-Item Short-Form Health Survey (SF-36), and the Functional Assessment of Cancer Therapy-Breast (FATC-B). The secondary outcomes include cardiopulmonary endurance, upper limb strength, lower limb strength, and skinfold thickness.

**Discussion:**

The importance of postoperative exercise rehabilitation for breast cancer has been gradually accepted by more and more doctors and patients, but further research and development of simple and practical means of exercise rehabilitation are necessary. Remote qigong intervention for breast cancer patients via the Internet will be a great alternative.

**Trial registration:**

Chinese Clinical Trial Registry ChiCTR1900027989. Registered on December 7, 2019.

## Background

Breast cancer is the most common malignant tumor in women worldwide and the second leading cause of cancer death in women. In recent years, with the continuous improvement of medical technology, the number of breast cancer survivors continues to grow. However, postoperative breast cancer patients often suffer from complications such as pain, limited upper limb activity, fatigue, obesity, premature menopause, and lymphedema, which seriously reduces the quality of life of patients. Therefore, more and more attention has been paid to the rehabilitation of postoperative breast cancer patients [1, 2].

Previous studies have revealed that exercise intervention will reduce mortality risk in breast cancer patients and improve the quality of life, physical function, and social cognition of survivors after breast cancer surgery [3, 4], and has a certain healing effect on patients after breast cancer surgery [5, 6]. Nevertheless, traditional exercise intervention failed to attract participation and improve the rehabilitation effect because of distance, time consumption, money, transportation, and so on. With the development of modern network technology, remote intervention has become possible. Studies have verified the effectiveness of remote intervention for breast cancer patients after surgery.

Qigong is a form of exercise focusing on physical activity, breathing, and psychological regulation, and a psychosomatic therapy [7]. Previous studies have shown that qigong exercise can improve the fatigue perception and sleep quality of breast cancer patients undergoing postoperative chemotherapy, reduce the risk of lymphedema, and improve their quality of life and physical fitness [8]. However, there is still a lack of research on the effect of remote exercise qigong intervention on postoperative recovery of breast cancer patients and it is also unclear whether qigong intervention is more advantageous than the conventional intervention combination of aerobic exercise and resistance exercise. This study is intended to verify the effect of long-range qigong intervention on the improvement of quality of life and physical fitness in breast cancer patients by means of randomized controlled trials and to compare it with conventional exercise intervention methods.

### Objectives

1. To explore whether Internet remote intervention can be used as a simple and practical means of exercise rehabilitation through experiments
2. To explore whether remote qigong intervention can improve the quality of life and physical quality of breast cancer patients

## Design and methods

### Design

**Fig. 1:**
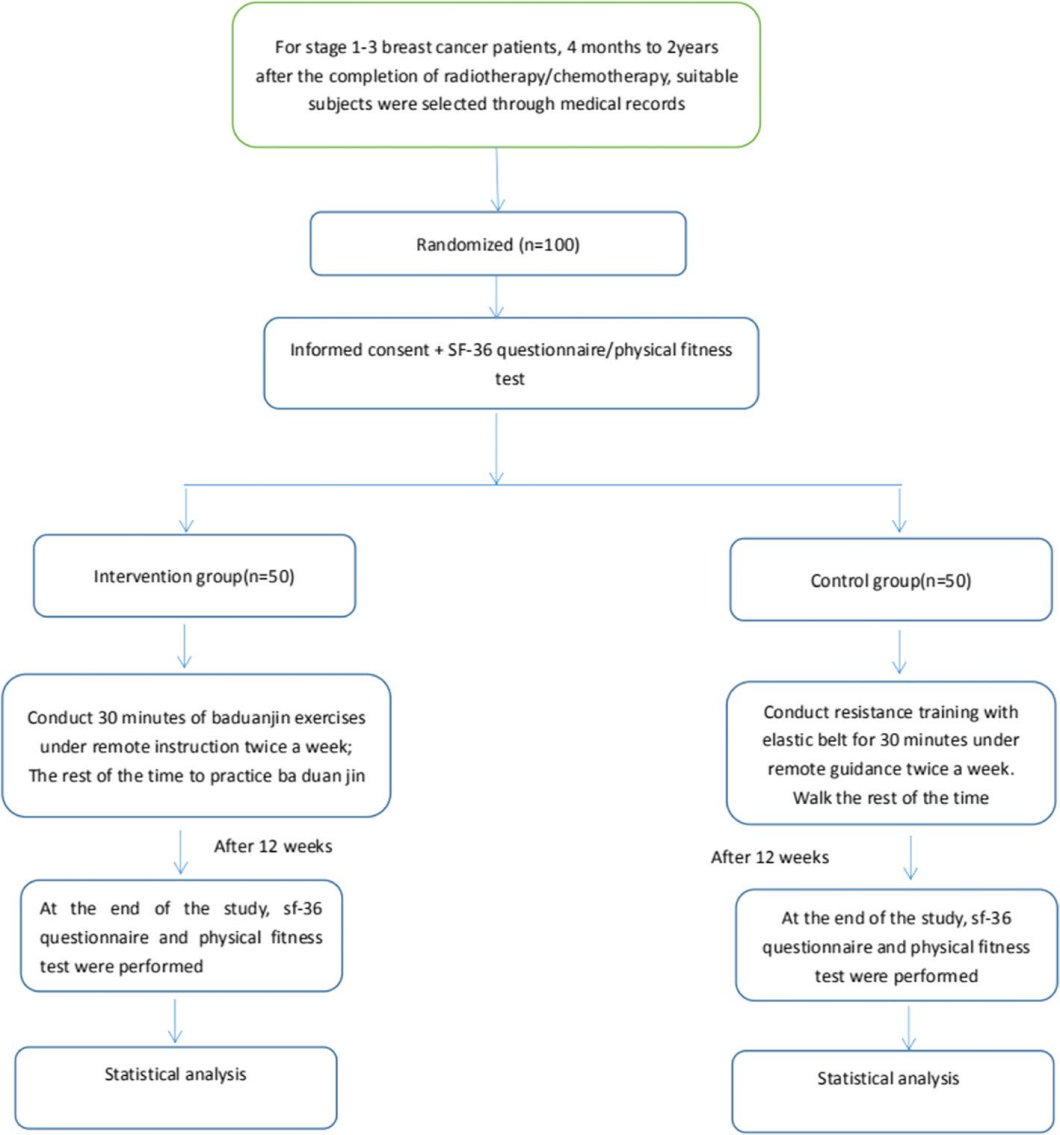
The flow chart of the trial design

This study is an RCT with an intervention time of 12 weeks. Trial participants will be recruited starting in February 2019 and the data will be analyzed after the sample size is achieved according to the study design. The flow chart of the study design is shown in Fig. 1.

### Subject recruitment

#### Inclusion criteria

1. Female breast cancer patients (stage I–III), aged over 18 years
2. No recurrence and no tumor metastasis
3. Radiotherapy/chemotherapy was completed within 4–18 months after surgery

#### Exclusion criteria

1. Language disorders or communication disorders
2. Serious exercise contraindications, such as cardiovascular or articular problems
3. Having any other serious illness or mental illness
4. Having received qigong exercise or other planned exercises
5. Unable to use the Internet by oneself or with the help of others

#### Drop out criteria

1. There were recurrence and tumor metastasis during the intervention
2. Subjects who cannot insist on receiving intervention for some reason

#### Recruiting methods

This study intends to recruit 100 subjects from Shandong province who meet the above standard through doctor recommendation or through newspapers, TV, and the Internet.

1. Planning the recruitment schedule and confirming the source of subjects
2. According to the inclusion and exclusion criteria, carefully check the database (prescription records, medical records), medical history cards, previous trials, and local epidemiological data of the disease
3. formulate the plan and method of contact with the subject, including the preparation of necessary data, videos, and pictures to help the subject understand the purpose of the test, and the design of the scheme
4. formulate the method of obtaining the informed consent form signed by the subject or the subject’s parents
5. Planning costs

### Subject grouping

Participants were briefed on the research and informed of the benefits and possible risks of clinical trials. They volunteered to participate in the study and completed an informed consent form. Subjects could withdraw from the study at any time. After the completion of informed consent, subjects were assigned to either the qigong group or the regular exercise rehabilitation group by way of a random number table. Both groups received supervised exercise twice a week and were encouraged to volunteer for 3–4 unsupervised exercise sessions per week.

### Allocation

#### Allocation concealment mechanism

The researchers who randomly assigned the sequence and determined the eligibility of the subjects were not the same person. Secondly, the research manager who generates and saves the randomly assigned sequence is the person who does not participate in the test. The treatment plan accepted by each subject was generated by the generated random distribution sequence and put into a sealed, light-tight envelope in sequence. Only when the qualified subjects agree to enter the test can the envelope be opened, and the subjects can accept the corresponding treatment measures.

### Implementation

After completing the baseline survey and physical fitness test, the computer random table generated by SPSS16.0 software was used for randomization. The eligible patients were randomly divided into an experimental group and control group, with a ratio of 1:1.

### Qigong group

Subjects in the qigong group conducted the Baduanjin through live video streaming under the guidance of professors and professionals. Baduanjin is one of the most widely circulated qigong methods, which is a traditional sport with a combination of physical activity, breathing, and psychological adjustment. The Baduanjin consists of eight movements, most of which are based on the horse stance combining movements of the upper limbs, head and neck, and spine, as well as organized breathing. In the first week, Baduanjin was taught by qualified qigong teaching staff through remote video, allowing the subjects to learn to master all movements of Baduanjin. Starting from week 2, subjects, led by a professional, performed Baduanjin twice a week, 30 min each, including 5 min of warming-up and cooling-down activities before and after the exercise. Participants were encouraged to actively practice Baduanjin during the rest of the time. Subjects were required to report their practice experience, possible adverse reactions, and side effects on a weekly basis, either orally or in writing. Subjects who exited the researcher were asked to explain the reason for the exit.

### Regular exercise rehabilitation group

The control group adopted a combination exercise of walking and resistance training [8]. Subjects underwent a 30-min resistance band training twice a week with a professional video tutorial, including 5 min of warm-up and cooling-down activities before and after the exercise. Subjects were encouraged to do walking exercise for the rest of the time, and the intensity of walking was determined based on pre-measured levels of aerobic capacity. The resistance load was determined based on the level of muscle strength measured in advance.

### Participant timeline

**Fig. 2:**
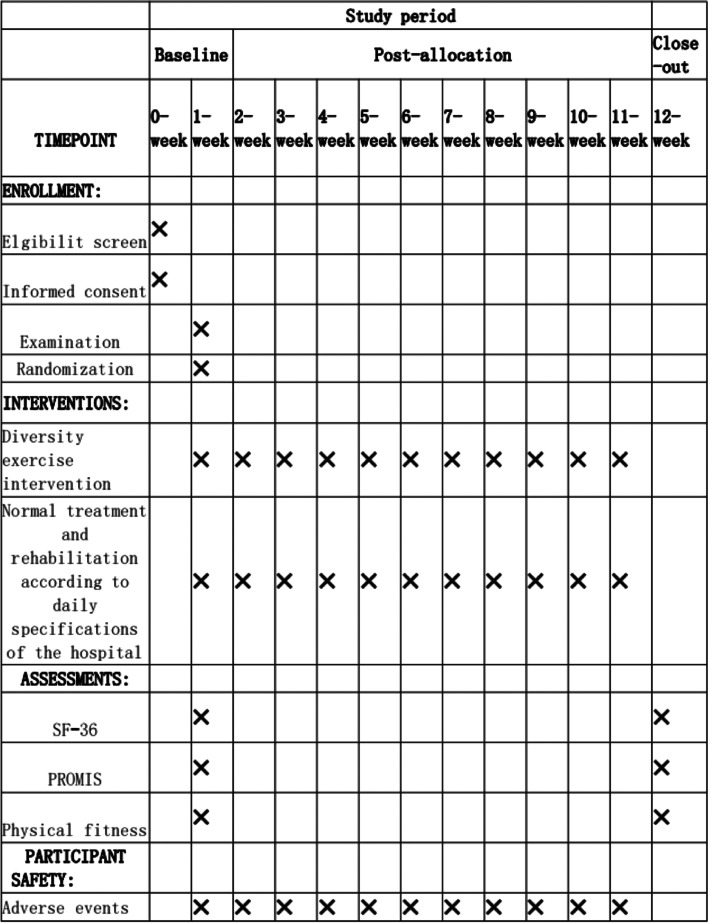
Recommendations for Interventional Trials (SPIRIT) diagram of enrolment, treatment, and assessments over time

Figure 2 shows details on the schedule of enrollment, interventions, and assessments.

### Outcomes

All data were measured and recorded 12 weeks after the baseline and intervention. The primary outcomes are quality of life which will be evaluated by using a 36-Item Short-Form Health Survey (SF-36) and Functional Assessment of Cancer Therapy-Breast (FATC-B), and the second indicator is the level of physical fitness, including cardiopulmonary endurance, muscle strength, and flexibility.

### Quality Of Life (QOL)

#### SF-36

Sf-36 [9, 10] is a general scale for evaluating health status, and its reliability and validity have been well recognized. The questionnaire included eight dimensions: physiological function (PF), role physical (RP), bodily pain (BP), general health (GH), vitality (VT), social function (SF), role emotional (RE), and mental health (MH).

#### FACT-B (Version 4.0)

FACT-B (version 4.0) [11, 12] is a targeted scale of quality of life for breast cancer survivors. It is recognized for good reliability, validity, and reactivity. FACT-B (version 4.0) includes five dimensions: physiological well-being (7 items), social/family well-being (7 items), emotional well-being (6 items), functional well-being (7 items), and additional concerns (9 items).

### Outcomes of physical fitness

#### Cardiopulmonary endurance

Bruce scheme was adopted and a cycle ergometer was used for testing.

#### Upper-limb strength test (Dumbbell Brachiocyrtosis Test)

Subjects were asked to sit on a 44CM high-backed chair placed against the wall, hold a 5-pound dumbbell, and extend their arms to the side of the chair naturally. Then they were asked to bend their arms upward to the maximum extent, then gradually swivel them outward to the palm up, and then put their arms back to the side of the chair. The number of repetitions within 30 s was recorded.

#### Lower-limb strength test (Sitting and Standing test)

For safety, the straight-back chair (or folding chair) was placed against the wall. Subjects sat in the middle of a chair with their feet shoulder-width apart, slightly forward and backward, and crossed arms close to the chest. During the test, the subjects stood up completely, then sat down completely. The number of times the subjects sat down in a chair within 30 s was recorded. For safety or requirement, the tester can use the arm for protective assistance [13].

#### Body fat composition

The percentage of body fat will be measured by the skinfold thickness method. The measurement included the triceps, anterior suprailiac, and thigh [14]. Bring the measured data of fold thickness (mm) into the corresponding body density formula to calculate the body density value, and then bring the body density value into the Siri or Brozek prediction formula to calculate the body fat percentage.

#### Flexibility (Hand Backward Stretch Test)

The subject’s hand was curled around the ipsilateral shoulder, stretching as far as possible into the middle of the back, with the palm close to the body. Subjects put the other hand behind the back, palm outward, stretched as far up as possible, and tried to touch or overlap the middle finger of both hands. The distance between the two middle fingers was measured twice in the vertical direction with an accuracy of 0.1 cm. If the fingertips just touch, the score is zero. If they were not touched, then a negative distance is gotten, like − 5 cm/ − 2 inches; If two fingers overlap, then a positive distance is acquired, such as + 2.5 cm/ + 1 inch; Finally, the best experimental results could be acquired. When the subject is in pain during the test, the test shall stop immediately.

### Data collection and management

In order to collect data in an accurate and scientific way, we trained personnel in charge of data measurement and collection with operational specifications. During the experimental data collection process, the subject’s physical condition was closely observed and if the subject appeared any discomfort, the test would immediately stop, and the subjects would timely consult a doctor.

Monitoring was carried out by personnel independent of investigators and sponsors, including checking all informed consent and verifying the integrity of all data and source data. They would collect data and record on standard reports, and when access was complete, all recorded data were transmitted to a web-based data system through multiple inputs. All errors were crossed out with the researcher’s signature and date. Any participant could withdraw from this study at any time for any reason.

### Statistical analysis

The experimental results used mixed-effect model [15] to compare the two sets of data. The random effect of the participants was evaluated, the data were adjusted according to the random group, and the relevant baseline variables were used to analyze the data without adjusting the multiple comparisons. The effect differences between the comparison groups were calculated (expressed as effect values), and the results were expressed as inter-group differences (95% confidence intervals). All analyses were performed using Stata 13.1 or the updated version.

### Blind method

After completing the baseline survey and physical fitness test, the computer random list generated by SPSS 16.0 software was used for randomization. Eligible patients were randomly divided into the experimental group and the control group, with a ratio of 1:1 [16]. The group numbers were enclosed in a carbon-free envelope. The envelopes were kept by the research manager, who was not directly involved in the recruitment or follow-up of any participants, and the group number would then be published. Different people would register as participants and be assigned for intervention, and the results were kept by the research designer until the end of the experiment [17].

### Sample size

The sample size was estimated based on our previous test results [18]. The sample size of this experiment was estimated by the non-inferior test. The non-inferiority margin was 6 and the standard deviation was 10.31. According to the formula, each group should have 44 participants by formulating the inspection level (0.025) and the power of test 1-β (0.2). During baseline inspection and follow-up, 50 participants in each group should participate in the experiment, given a 15% expulsion rate.

## Discussion

The effect of qigong intervention on postoperative rehabilitation of breast cancer has been confirmed by randomized controlled trials [9]. However, there are some problems in the traditional outpatient exercise rehabilitation, such as traffic, time consumption, and space occupation, which will affect the patients’ enthusiasm to participate in rehabilitation to a certain extent, and also increases the medical economic burden. The development of Internet technology makes it possible to guide the rehabilitation of remote exercise. Based on this, we designed a randomized controlled trial with a sufficient sample size to verify the rehabilitation effect of remote guidance intervention of qigong on postoperative breast cancer patients and to compare the effects of qigong intervention and traditional exercise rehabilitation.

In addition, most of the study of exercise rehabilitation takes the improvement of patients’ physical fitness level as the main research outcome, which neglects the patient’s subjective feeling and quality of life to some extent. Therefore, the quality of life of breast cancer patients after surgery is set as the first research indicator, followed by the related physical fitness outcomes.

Also, it is not clear whether conventional exercise intervention or qigong intervention can prevent recurrence in breast cancer patients. To answer this question, a follow-up study of larger samples and longer periods of time are necessary. Due to the limitation of research funding, The exercise intervention and follow-up time are relatively short which makes it hard to study the recurrence of tumors in depth. If possible, subsequent studies will look at whether qigong can prevent breast cancer recurrence.

## Abbreviations

Sf-36: The MOS 36-item Short Form Health Survey
PF: Physiological function
RP: Role physical
BP: Bodily pain
GH: General health
VT: Vitality
SF: Social function
RE: Role emotional
MH: Mental health
